# Genetic Variants Associated with FDNY WTC-Related Sarcoidosis

**DOI:** 10.3390/ijerph16101830

**Published:** 2019-05-23

**Authors:** Krystal L. Cleven, Kenny Ye, Rachel Zeig-Owens, Kerry M. Hena, Cristina Montagna, Jidong Shan, H. Dean Hosgood, Nadia Jaber, Michael D. Weiden, Hilary L. Colbeth, David G. Goldfarb, Simon D. Spivack, David J. Prezant

**Affiliations:** 1 Pulmonology Division, Department of Medicine, Montefiore Medical Center, Bronx, NY 10467, USA; rachel.zeig-owens@fdny.nyc.gov (R.Z.-O.); hilary.colbeth@fdny.nyc.gov (H.L.C.); david.goldfarb@fdny.nyc.gov (D.G.G.); simon.spivack@einstein.yu.edu (S.D.S.); david.prezant@fdny.nyc.gov (D.J.P.); 2 Pulmonology Division, Department of Medicine, Albert Einstein College of Medicine, Bronx, NY 10467, USA; 3 Department of Epidemiology and Population Health, Albert Einstein College of Medicine, Bronx, NY 10461, USA; dean.hosgood@einstein.yu.edu; 4 Fire Department of the City of New York, Bureau of Health Services, Brooklyn, NY 11201, USA; nadia.jaber@fdny.nyc.gov (N.J.); michael.weiden@nyulangone.org (M.D.W.); 5 Pulmonary & Critical Care Division, Department of Medicine, NYU School of Medicine, New York, NY 10016, USA; kerry.hena@nyulangone.org; 6 Department of Genetics, Albert Einstein College of Medicine, Bronx, NY 10461, USA; cristina.montagna@einstein.yu.edu (C.M.); jidong.shan@einstein.yu.edu (J.S.); 7 Department of Pathology, Albert Einstein College of Medicine, Bronx, NY 10461, USA; 8 Molecular Cytogenetic Core, Albert Einstein College of Medicine, Bronx, NY 10461, USA

**Keywords:** sarcoidosis, World Trade Center, 9/11, genetics, firefighters, FDNY

## Abstract

Sarcoidosis is a systemic granulomatous disease of unknown etiology. It may develop in response to an exposure or inflammatory trigger in the background of a genetically primed abnormal immune response. Thus, genetic studies are potentially important to our understanding of the pathogenesis of sarcoidosis. We developed a case-control study which explored the genetic variations between firefighters in the Fire Department of the City of New York (FDNY) with World Trade Center (WTC)-related sarcoidosis and those with WTC exposure, but without sarcoidosis. The loci of fifty-one candidate genes related to granuloma formation, inflammation, immune response, and/or sarcoidosis were sequenced at high density in enhancer/promoter, exonic, and 5’ untranslated regions. Seventeen allele variants of human leukocyte antigen (HLA) and non-HLA genes were found to be associated with sarcoidosis, and all were within chromosomes 1 and 6. Our results also suggest an association between extrathoracic involvement and allele variants of HLA and non-HLA genes found not only on chromosomes 1 and 6, but also on chromosomes 16 and 17. We found similarities between genetic variants with WTC-related sarcoidosis and those reported previously in sporadic sarcoidosis cases within the general population. In addition, we identified several allele variants never previously reported in association with sarcoidosis. If confirmed in larger studies with known environmental exposures, these novel findings may provide insight into the gene-environment interactions key to the development of sarcoidosis.

## 1. Introduction

Sarcoidosis is a multisystem granulomatous disease that presents with pulmonary involvement in 90% of cases and extrapulmonary manifestations, mostly skin, in 10% of patients [1]. The annual age-adjusted incidence in the United States (U.S.) between 1946–2013, in a mostly Caucasian cohort, was 10 per 100,000 (9.4 in males and 10.5 in females) [2]. Between 1985 and 1998, firefighters in the Fire Department of the City of New York (FDNY) had an annual incidence of sarcoidosis somewhat higher than the white male general population at 12.9 per 100,000 and many-fold higher than an internal control group of emergency service workers [3]. The incidence doubled following the World Trade Center (WTC) attacks on September 11, 2001 (9/11), giving an age-adjusted incidence rate of 25 per 100,000 in mostly Caucasian male WTC-exposed FDNY firefighters between 2002–2015 [4].

Since the disorder clusters in families, genetic factors are thought to play an important role in sarcoidosis pathogenesis, presumably through mechanisms of immunologic response to antigenic or other triggering exposures. A recent population-based study suggests a genetic risk factor for sarcoidosis in a large Swedish cohort, where a 3.7-fold increase in risk of sarcoidosis was observed in those with first-degree relatives with the disease. The risk further increased in those with relatives with Lofgren’s syndrome or with ≥1 relative with sarcoidosis [5]. A case-control etiologic study of sarcoidosis (ACCESS) found a five-fold increase in siblings of those with sarcoidosis [6].

We selected several candidate genes for genetic analysis based on their function encoding proteins involved in granuloma formation, innate and immune recognition, and inflammation. Several have been implicated in genetic studies of sarcoidosis such as the highly polymorphic human leukocyte antigen (HLA) genes. HLA genes encode the major histocompatibility complex (MHC) in humans and are involved in immunologic recognition and regulation. Within various racial groups, allele variations within HLA genes are associated with sarcoidosis. Examples include the HLA-DRB1*1101 allele in both Caucasians and African Americans [7], HLA-DRB1*1501, -*03 and -*0402 specifically in European Americans [7,8,9], HLA-DRB1*1201 and -*0302 in African-Americans [7,10]. The HLA-DQB1 alleles HLA-DQB1-*0402, and -*0503 are associated in Asian Indians [7] and HLA-DQB1-*0602 in a Dutch cohort [8]. Several other non-HLA genes have been associated with sarcoidosis, such as prostaglandin G/H synthase/cyclooxygenase (PTGS2/COX2), neurogenic locus notch homolog 4 (NOTCH4), nucleotide-binding oligomerization domain-containing protein 2 (NOD2), and Butyrophilin-like 2 (BTNL2) [11,12,13,14,15].

The current case-control study is the first to examine genetic features associated with WTC-related sarcoidosis. Compared to other genetics studies of sarcoidosis, our study uniquely includes a baseline sarcoidosis rate, a known time point of exposure (9/11), and extensive, systematized clinical follow-up for over 14 years. The objective of this study is to explore the genetic characteristics of WTC-associated sarcoidosis and understand its similarities to and differences from sporadic sarcoidosis cases without a clear environmental exposure. 

## 2. Materials and Methods

### 2.1. Study Population

The source population for the cases and controls was WTC-exposed firefighters. Recruitment of sarcoidosis cases for this study was part of a larger study that explored the clinical course of firefighters with post-9/11 sarcoidosis between the time of diagnosis and 14 years after 9/11 [16]. As outlined in Hena, et al (2018), cases were identified as WTC-exposed firefighters with WTC-related sarcoidosis if they had normal chest imaging prior to 9/11 and then post-9/11 radiographic findings consistent with sarcoidosis [16]. Extra-pulmonary organ involvement was determined from study questionnaires and medical records and was defined according to the World Association of Sarcoidosis and Other Granulomatous Diseases (WASOG) criteria [17].

Controls were randomly selected from the source population and were matched to cases based on race, age on 9/11, smoking history, and WTC-exposure history. WTC-exposure history was determined by arrival time to the WTC site: arrival group 1 (arriving the morning of 9/11) being the group with the highest dust cloud exposure and arrival group 4 (arriving between 9/13–9/24) having the least dust cloud exposure [18]. Controls also had normal chest radiographs at the time of the study.

The final study population included 55 cases and 100 controls. All cases and controls had peripheral blood drawn in 2015–2016 and completed a questionnaire at the time of blood draw to aid with matching on clinical characteristics and WTC-exposure history. The study was approved by the Montefiore Medical Center/Albert Einstein Institutional Review Board (2014-4291). All study participants provided written informed consent.

### 2.2. Genotyping

Peripheral blood mononuclear cells were obtained from cases and controls for sequencing analysis. A panel of 51 candidate genes were pre-selected based on their function encoding proteins involved in granuloma formation, innate and immune recognition, inflammation, and sarcoidosis. The loci of targeted candidate genes were sequenced using a custom designed sequencing panel for IonTorrent Ampliseq technology. The custom sequencing panel contained 1193 amplicons spanning all known exons, splice junctions and promoter regions of the genes. Promoter regions were defined as 2 kb upstream from the transcriptional start site. The panel overall coverage was over 90% for 422 exons (Supplementary Table S1). One exception was HLA-A, which had 41% coverage, but is known to be associated with low coverage in prior design assays as well. The sequencing panel was designed for the analysis of amplicons of 125–375 bp using the Ion AmpliSeq technology and was suitable for sequencing using the Ion Proton Sequencer as previously reported [19].

DNA was extracted using the QuickGene 610 system and the whole blood kit (Kurabo #DB-L), and quality verified and quantitated by the NanoDrop^TM^ 1000 Spectrophotometer (Thermo Fischer Scientific, Waltham, MA, USA). The Qubit^®^ dsDNA HS Assay Kit (Life Technologies, Carlsbad, CA, USA) was used to determine the concentration of each sample. Approximately 20 ng of DNA was used to generate libraries for sequencing. When not in use, samples were stored at −20 °C. For each sample, a PCR reaction was set up according to the manufacturer’s protocol using 20 ng of genomic DNA, two separate primer pools, and 16 amplification cycles. Following amplification, each sample was treated with FuPa reagent and ligated to a uniquely barcoded adapter to enable sample multiplexing. Libraries were then purified using the 1.5X Agencourt AMPure XP (Beckman Coulter Inc, Brea, CA, USA) kit. Amplification products from each primer pool were quantified individually using the KAPA Library Quantitation kit (Roche, Mannheim, Germany) and then pooled together. Template preparation was performed using the Ion OneTouch 2 system and the Ion PI Template OT2 200 kit v2 (Thermo Fisher Scientific, Waltham, MA, USA), according to the manufacturer’s protocol. All libraries were sequenced on the Ion Proton sequencer using the Ion PI chip and the Ion PI Sequencing 200 kit v2 (Thermo Fisher Scientific, Waltham, MA, USA) to generate 200bp single ended sequencing.

For the identification and reporting of germline variants, we used a custom validated bioinformatic pipeline for the identification of SNPs as well as insertions and deletions (INDELs) included in the target panels [20,21,22]. The sequence reads obtained from the samples were aligned to the human reference genome (hg19-Genome Reference Consortium GRCh37) using the Ion Torrent Suite and then processed by the Torrent Variant Caller for variant calling. The generated BAM files were next imported into the Ion Reporter Software^TM^ (Thermo Fisher Scientific, Waltham, MA, USA) for filtering and annotation. 

### 2.3. Statistical Analysis

#### 2.3.1. Common Variants

We identified 909 variants (SNPs or INDELs) with minor allele frequency (MAF) ≥0.05 and *p*-value for Hardy-Weinberg Equilibrium Test > 0.0001. The variants were classified by their genomic locations to four categories: exon, intron (likely intron/exon boundaries), upstream, and 5’untranslated region (5’UTR). For those in an exonic region, they were further classified by their function as synonymous, nonsense, missense, or frameshift (Supplementary Tables S2 and S3). For each of these common variants, Pearson Chi-square test was used to compare the genotype frequencies between the 55 cases and 100 controls that were successfully sequenced (see Supplementary Table S4). To verify the robustness of our results, Fisher’s Exact Tests were also performed. For each of the variants, odds ratios were estimated using unconditional logistic regression under the co-dominant model.

We conducted two secondary analyses. First, we explored the interaction between each common variant and degree of WTC exposure, measured as arrival time to the WTC site. For each variant, we applied unconditional logistic regression under the co-dominant model with sarcoidosis as the outcome and the genotypes, WTC exposure group and their interaction term, as the explanatory variables. Likelihood ratio tests are applied to compare two models with and without the interaction term between the genotype and degree of WTC exposure. We also conducted a secondary analysis in which we associate the genetic variants with extrathoracic sarcoidosis. In these analyses, for each variant we applied unconditional logistic regression under the co-dominant model with extrathoracic sarcoidosis as the outcome and the genotypes as the explanatory variable.

#### 2.3.2. Rare Variants

For variants whose MAF < 0.05, for each individual, the total number of rare alleles located in each gene were counted. We then compared the number of rare alleles between the cases and controls for each gene by each genomic location category (exon, intron, upstream, or 5’untranslated region) using two-sample T-tests. Furthermore, we counted rare variants with MAF < 0.01 across all target genes by the above-mentioned categories by genomic location and function, and then performed two-sample t-tests comparing the cases and controls for each of the categories.

## 3. Results

Final genetics analyses were completed on 55 cases and 100 controls. All but one case (54/55) and one control (99/100) self-identified as Caucasian, the remaining one case and one control self-identified as African American. The cases and controls were well matched, with at least one control for each case (Table 1).

Organ involvement for the 55 cases included 47/55 (85.5%) with biopsy proven sarcoidosis. The 8/55 (14.5%) without biopsy results had their imaging and organ involvement reviewed by two pulmonologists to confirm consistency with sarcoidosis [16]. As outlined in Table 2, there was a decrease in intrathoracic involvement from the time of diagnosis to the time of the blood draw (mean eight-years post diagnosis) from 95% to 47% and an increase in extrathoracic involvement, especially cardiac and joint.

Within the 51 candidate genes analyzed, we identified 3619 total number of variants. A total of seventeen common variants were found to be associated with sarcoidosis with Chi-Squared *p*-value < 0.01 (Table 3). All of the 17 variants were within chromosomes 1 and 6. Multiple variants were in HLA genes such as HLA-C, HLA-DRB1, HLA-DQB1, HLA-DPA1, and HLA-DPB1. There was a strong association with sarcoidosis within exonic regions of the HLA-DQB1 gene, represented by rs1049133 and rs1049130, two SNPs 12bp apart (ORs = 2.56 and 1.90, respectively). Two variants upstream from an intronic/exonic border region of HLA-DQB1 were also significantly associated with sarcoidosis, rs4516985 and rs9274614 (ORs = 1.74 and 2.49, respectively). In addition, several genetic variants within or near non-HLA genes were also significantly associated with sarcoidosis: BTNL2, PTGS2/COX2, and PACERR (PTGS2 Antisense NFKB1 Complex-Mediated Expression Regulator RNA). SNPs in a non-coding region of BTNL2, rs2076525 and rs2076524, were significantly associated with sarcoidosis (OR = 1.71). In addition, rs2076523, representing a missense mutation within a BTNL2 coding region was associated (OR = 1.97). Upstream from PTGS2/COX2 gene, rs20417 was also associated with sarcoidosis cases in our cohort (OR = 1.79).

In our secondary analysis, we did not find statistical evidence of an interaction between common variants and the degree of WTC exposure. We also found no statistical significance when we compared the number of rare variants between the cases and controls.

Our results also suggest an association between extrathoracic involvement and genetic variants within several HLA and non-HLA genes: HLA-B, PTGS2/COX2, PACERR, NOTCH4, NOD2, and ITGAE (Integrin Subunit Alpha E) (Table 4). Genetic variants associated with extrathoracic cases were found on chromosomes 1 and 6, similar to the loci associated with all sarcoidosis cases, and were also found on chromosomes 16 and 17. On chromosome 1, rs2066826 represents an intronic region/exonic border of PTGS2/COX2 associated with sarcoidosis, and specifically, extrathoracic disease in this cohort (OR 1.88 and 1.45, respectively). As seen in all sarcoidosis cases with rs20417, another variant only a few hundred base pairs away and downstream from PACERR, is rs689466, for which the allele C was more common in those with extrathoracic disease. A genetic variant within an HLA-B, rs2276448, was also associated with extrathoracic sarcoidosis. In addition, on chromosome 6, a locus upstream from NOTCH4, rs3134929, was associated with extrathoracic disease. And in chromosome 16 and 17, three variants were associated with extrathoracic involvement: two SNPs within chromosome 16, rs2066843 and rs2066842, both within exonic regions of NOD2; and one within chromosome 17, rs220465, representing a genetic variant within the intronic region/exonic border of ITGAE. As shown in Table 2, cardiac and joint involvement were common, but our cohort size was too small to identify genetic variants associated with any specific extrathoracic organ manifestation. We also found no association between the degree of exposure and extrathoracic organ involvement.

## 4. Discussion

Sarcoidosis has been identified in all WTC cohorts (FDNY, general responders other than FDNY, the survivor population and the WTC Registry), the most extensively described being that from FDNY [4,16,23,24,25,26]. In our current FDNY case-control study, we examine genetic characteristics in our cases as compared with our WTC-exposed controls without sarcoidosis. Seventeen allele variants of HLA and non-HLA genes were found to be associated with sarcoidosis with *p*-value < 0.01 and all were within chromosomes 1 and 6. Our results also suggest an association between extrathoracic involvement and allele variants of HLA and non-HLA genes found not only on chromosomes 1 and 6, but also on chromosomes 16 and 17. Comparing our findings to published studies of sporadic sarcoidosis, we found similarities between genetic variants with WTC-related sarcoidosis and those reported in studies of sarcoidosis without known environmental exposures. In addition, we identified several allele variants not previously reported to be in association with sarcoidosis. If confirmed in larger studies with known environmental exposures, these novel findings may provide insight into the gene-environment interactions key to the development of sarcoidosis.

For example, our data are consistent with a prior study showing an association between SNP rs20417, in a non-coding region of the PTGS2/COX2 gene on chromosome 1, and sarcoidosis in a UK and Austrian Caucasian cohort without known environmental exposure [11]. This allele variant, described using prior nomenclature -765C (-765 is the nucleotide base pair position and C is the base pair), was found to be more often present in sarcoidosis subjects with severe pulmonary disease (poor lung function or extensive fibrosis). The authors also created lung fibroblast cell lines that were stimulated by transforming growth factor (TGF)-β1. They found that the cell lines with the -765C (currently named rs20147) produced little to no prostaglandin E2 (PGE2), an important inhibitor of the proliferation of fibroblasts. The loss of this lung protective inflammatory mediator could explain its relationship to more severe forms of pulmonary sarcoidosis. As eloquently described by Valentonyte and colleagues (2005), allele variations in BTNL2 impact the negative T-cell regulation function of BTNL2 [14]. Thus, the dysregulation of T-cell function from some BTNL2 mutations may explain its relationship to sarcoidosis. Interestingly, Akers and colleagues (2011) determined there was strong linkage disequilibrium between rs9274614, an allele significantly associated with sarcoidosis in our cohort, and several HLA-DQB1*06 alleles [27]. Another study found an association between the HLA-DQB1*0601 allele and cardiac sarcoidosis in a Japanese cohort [28]. Although our sample size was too small to identify specific alleles associated with cardiac sarcoidosis, the known association between the SNP rs9274614 and an allele associated with cardiac sarcoidosis, HLA-DQB1*0601, is one of interest in this cohort given the significant number of participants with cardiac sarcoidosis.

Except as noted above, the genetic variants associated with sarcoidosis in our WTC-exposed cohort have not previously been reported. Several of the SNPs identified in our cohort as associated with WTC-related sarcoidosis have been associated with other diseases in cohorts without known environmental exposures. Perhaps the most frequently investigated is rs20417 in PTGS2/COX2. Studies have identified its association with carotid-calcified plaque in those with diabetes [29], protection against myocardial infarction and stroke [30], as well as asthma [31]. In addition, SNP rs1126513, significantly associated with WTC-related sarcoidosis in our cohort, was found to be associated with ankylosing spondylitis [32]. 

The main strength of this study was its reliance on a rigorously characterized prospective cohort, with a clearly defined time of exposure (9/11). One limitation of this study is that it was focused on candidate genes already identified in sarcoidosis studies from the general population without WTC exposure. Additionally, the identification of novel SNPs may be partially due to our high-density sequencing which evaluated almost every base pair of these candidate genes. The main limitation, however, is that this study includes a relatively small sample size. Our statistical power was limited to detect only sizable effects. At significance level 0.01, we had 0.8 power to detect an odds ratio of 2.3 at variants with MAF = 0.5 and an odds ratio of 4 at variants with MAF = 0.05; we had 0.2 power to detect an odds ratio of 1.5 at variants with MAF = 0.5 and an odds ratio of 2 at variants with MAF = 0.05. Additional power would be obtained if this study was expanded to include all of the various WTC health program cohorts to allow for both replication of the main effects observed, and to allow for gene-environment analyses. This could potentially allow for more robust causal inferences to be made regarding the etiology of sarcoidosis, and whether there are unique differences in those with extrathoracic disease, particularly cardiac and joint involvement.

## 5. Conclusions

The incidence of sarcoidosis was increased after the collapse of the WTC towers and this is best demonstrated in the FDNY cohort where there was clear evidence that these cases were new in onset after 9/11 [4,16]. Our findings suggest that the genetic characteristics of WTC-related sarcoidosis are similar to the sporadic cases found in the general population without known environmental exposures. These data suggest the granulomatous disease observed post-9/11 and originally termed “Sarcoid-Like” Granulomatous Disease (SLGD) is better described as WTC-related Sarcoidosis. Our findings, however, suggest that there are several novel allele variants (SNPs) which may be uniquely associated with WTC-related sarcoidosis and with extrathoracic disease. These novel findings may provide some insights into the unique gene-environment interactions key to the development of sarcoidosis in this population, among others. Future studies should focus on increasing the power of our findings by combining sarcoidosis cases from all WTC health program cohorts and comparing them to matched controls with non-WTC associated sarcoidosis.

## Figures and Tables

**Table 1:** Demographics of WTC Study Population.

Demographics	Case Characteristics		Control Characteristics		*p*-Value
	*N*	%	*N*	%	
**Total**	55	100	100	100	
**Arrival group**					0.99
**Morning of 9/11**	12	21.8	22	22	
**Afternoon of 9/11**	24	43.6	46	46	
**Day 9/12**	15	27.3	25	25	
**Day 9/13–9/24**	4	7.3	7	7	
**Race**					1.00
**White**	54	98.2	99	99	
**African-American**	1	1.8	1	1	
**Smoking status at time of blood draw**					0.66
**Never**	40	72.7	72	72	
**Former**	14	25.5	23	23	
**Current**	1	1.8	5	5	
**Age on 9/11, years, median [IQR]**	36.8 [32.7–39.0]		35.4 [30.2–39.3]		0.29
**Age at sarcoidosis diagnosis, median [IQR]**	43.0 [38.2–46.6]				--
**Time to diagnosis post 9/11, median [IQR]**	6.7 [3.3–9.6]				--

IQR: interquartile range.

**Table 2:** Sarcoidosis organ involvement in WTC genetics study population.

Organ Involvement	Study Cases (*N* = 55)	
	At Diagnosis	Time of Blood Draw ^a^
Bone marrow	0	0
Bone/joint	3	8
Calcium	0	0
Cardiac	0	9
Ear/nose/throat	0	1
Extrathoracic lymph nodes	0	0
Eyes	3	3
Intrathoracic involvement by CT Imaging	52	26
Kidney	1	0
Liver	1	1
Muscle	0	0
Nervous system	0	1
Other organs	0	0
Salivary	0	0
Skin	1	1
Spleen	2	2

^a^ All cases had complete data regarding organ involvement at diagnosis and blood draw with the following exceptions: chest CT scan at diagnosis (*N* = 52), and at blood draw (*N* = 53); muscle at diagnosis (*N* = 54), and at blood draw (*N* = 54); spleen at diagnosis (*N* = 54), and at blood draw (*N* = 53); cardiac at blood draw (*N* = 53); and ear/nose/throat at diagnosis (*N* = 51), and at blood draw (*N* = 53).

**Table 3:** Genetic variants most associated with sarcoidosis.

Gene	Position(hg19)	dbSNP	Alleles *	Risk Allele	Chi-sq *p*-Value	Fisher’s *p*-value	OR **
**PTGS2/COX2**	chr1:186645927	rs2066826	T/C	C	0.002	0.001	1.88
**PTGS2|PACERR ^⇞^**	chr1:186650321	rs20417	G/C	C	0.003	0.001	1.79
**HLA-C**	chr6:31239681	rs9264669	T/A	A	0.004	0.003	1.75
**BTNL2**	chr6:32370616	rs2076525	C/T	T	0.006	0.003	1.71
**BTNL2**	chr6:32370684	rs2076524	G/A	A	0.006	0.003	1.71
**BTNL2**	chr6:32370835	rs2076523	C/T	T	0.005	0.002	1.97
**HLA-DRB1**	chr6:32549424	rs112116022	T/C	C	0.003	0.002	5.21
**HLA-DQB1**	chr6:32629847	rs1049133	G/A	A	0.004	0.003	2.56
**HLA-DQB1**	chr6:32629859	rs1049130	G/A	A	0.006	0.004	1.90
**HLA-DQB1**	chr6:32635632	rs4516985	G/A	A	0.004	0.005	1.74
**HLA-DQB1**	chr6:32635846	rs9274614	G/C	C	0.005	0.004	2.49
**HLA-DPA1|HLA-DPB1 ^⇞^**	chr6:33048457	rs386699868 rs1126504	G/C	G	0.007	0.006	1.80 ^†^
**HLA-DPA1|HLA-DPB1 ^⇞^**	chr6:33048466	rs386699869 rs1126511 rs1126513	TT/GG	TT	0.007	0.007	1.66 ^†^
**HLA-DPA1|HLA-DPB1 ^⇞^**	chr6:33049211	rs928976	T/C	T	0.007	0.008	1.74 ^†^

⇞ indicates that SNP overlaps part of both genes; * listed as alternative allele/reference allele; ** ORs are calculated under the co-dominant model and are relative to the alternative allele unless specified with † which is in relation to the reference allele. This was done to maintain the OR as a comparison between the risk allele and non-risk allele.

**Table 4:** Genetic variants associated with extrathoracic organ involvement.

Gene	Position(hg19)	dbSNP	Alleles *	Risk Allele	Chi-sq *p*-Value	Fisher’s *p*-Value	OR **
**PTGS2**	chr1:186645927	rs2066826	T/C	C	0.009	0.012	1.45
**PACERR**	chr1:186650751	rs689466	C/T	C	0.006	0.016	2.33 ^†^
**HLA-B**	chr6:31323020	rs2276448	C/T	T	0.008	0.003	2.13
**NOTCH4**	chr6:32192107	rs3134929	G/C	G	0.009	0.012	2.39 ^†^
**NOD2**	chr16:50744624	rs2066842	T/C	T	0.010	0.019	2.00 ^†^
**NOD2**	chr16:50745199	rs2066843	T/C	T	0.010	0.019	2.21 ^†^
**ITGAE**	chr17:3637915	rs220465	T/C	T	0.005	0.006	1.08 ^†^

* listed as alternative allele/reference allele; ** ORs are calculated under the co-dominant model and are relative to the alternative allele unless specified with † which is in relation to the reference allele. This was done to maintain the OR as a comparison between the risk allele and non-risk allele.

## Supplementary Materials

The following are available online at https://www.mdpi.com/1660-4601/16/10/1830/s1, Table S1: Candidate genes and exon coverage, Table S2: Location and function of gene variants associated with sarcoidosis, Table S3: Location and function of gene variants associated with extrathoracic organ involvement, Table S4: Genotype frequencies.
