# Supplementary material for: Genetic Variants Associated with FDNY WTC-Related Sarcoidosis
Source: Int J Environ Res Public Health. 2019 May 23;16(10):1830. doi: 10.3390/ijerph16101830 (PMC6572061; doi:10.3390/ijerph16101830)
Supplement: Supplementary file 1 [file ijerph-16-01830-s001.zip › ijerph-456356-supplementary-forxml/ijerph-456356-supplementary-forproof/ijerph-456356-supplementary.docx]

**Supplemental Table S1.** Candidate genes and exon coverage.

|  | Gene Name | Number of Exons | Coverage (%) | Promoter Included (2Kb Upstream TSS) |
| --- | --- | --- | --- | --- |
| 1 | ANXA11 | 15 | 100 | YES |
| 2 | BTNL2 | 6 | 100 | YES |
| 3 | C10orf67 | 5 | 100 | YES |
| 4 | CCDC88B | 27 | 95.7 | YES |
| 5 | CCL5 | 5 | 100 | YES |
| 6 | CCR2 | 3 | 100 | YES |
| 7 | CCR5 | 1 | 100 | YES |
| 8 | CD14 | 2 | 86.4 | NO |
| 9 | CFTR | 27 | 99.9 | NO |
| 10 | GREM1 | 4 | 100 | YES |
| 11 | HLA-A | 8 | 41.8 | YES |
| 12 | HLA-B | 7 | 77.1 | YES |
| 13 | HLA-C | 8 | 74.6 | YES |
| 14 | HLA-DPA1 | 4 | 100 | YES |
| 15 | HLA-DPB1 | 5 | 100 | YES |
| 16 | HLA-DQA1 | 4 | 86.6 | YES |
| 17 | HLA-DQB1 | 6 | 76.3 | YES |
| 18 | HLA-DRB1 | 8 | 79.1 | YES |
| 19 | IFNA17 | 1 | 100 | YES |
| 20 | IL10 | 5 | 100 | YES |
| 21 | IL18 | 5 | 98.1 | YES |
| 22 | IL1A | 6 | 100 | NO |
| 23 | IL23A | 4 | 100 | NO |
| 24 | IL23R | 10 | 100 | YES |
| 25 | IL4 | 4 | 100 | YES |
| 26 | IL6 | 5 | 100 | YES |
| 27 | IL7R | 8 | 100 | YES |
| 28 | ITGAE | 31 | 99.6 | YES |
| 29 | KDR | 30 | 100 | YES |
| 30 | MHC2TA | 21 | 98.9 | YES |
| 31 | MMP9 | 13 | 99.2 | YES |
| 32 | MRC1 | 30 | 99.5 | YES |
| 33 | MYD88 | 7 | 100 | YES |
| 34 | NOD2 | 12 | 93.8 | YES |
| 35 | NOTCH4 | 30 | 98.1 | YES |
| 36 | OS9 | 18 | 100 | YES |
| 37 | PTGS2 | 10 | 100 | YES |
| 38 | RAB23 | 6 | 100 | YES |
| 39 | SLC11A1 | 15 | 100 | YES |
| 40 | SPP1 | 7 | 100 | YES |
| 41 | TGFB1 | 7 | 100 | YES |
| 42 | TGFB2 | 8 | 92.9 | YES |
| 43 | TGFB3 | 7 | 98 | YES |
| 44 | TLR1 | 1 | 100 | YES |
| 45 | TLR10 | 2 | 100 | YES |
| 46 | TLR2 | 1 | 97.4 | YES |
| 47 | TLR4 | 5 | 100 | NO |
| 48 | TLR6 | 1 | 100 | YES |
| 49 | TNF | 4 | 100 | YES |
| 50 | VDR | 11 | 100 | YES |
| 51 | VEGFA | 15 | 99.1 | YES |

**Supplemental Table S2.** Location and function of gene variants associated with sarcoidosis.

| Gene | Position(hg19) | dbSNP | Location | Function |
| --- | --- | --- | --- | --- |
| PTGS2 | chr1:186645927 | rs2066826 | intronic |  |
| PTGS2\|PACERR | chr1:186650321 | rs20417 | upstream\|exonic_nc | non-coding |
| HLA-C | chr6:31239681 | rs9264669 | intronic |  |
| BTNL2 | chr6:32370616 | rs2076525 | intronic |  |
| BTNL2 | chr6:32370684 | rs2076524 | intronic |  |
| BTNL2 | chr6:32370835 | rs2076523 | exonic | missense |
| HLA-DRB1 | chr6:32549424 | rs112116022 | exonic | missense |
| HLA-DQB1 | chr6:32629847 | rs1049133 | exonic | synonymous |
| HLA-DQB1 | chr6:32629859 | rs1049130 | exonic | synonymous |
| HLA-DQB1 | chr6:32635632 | rs4516985 | upstream\|intronic |  |
| HLA-DQB1 | chr6:32635846 | rs9274614 | upstream\|intronic |  |
| HLA-DPA1\|HLA-DPB1 | chr6:33048457 | rs386699868 rs1126504 | utr5\|exonic | missense |
| HLA-DPA1\|HLA-DPB1 | chr6:33048466 | rs386699869 rs1126511 rs1126513 | utr5\|exonic | missense |
| HLA-DPA1\|HLA-DPB1 | chr6:33049211 | rs928976 | upstream\|intronic |  |

| indicates that location is between two genomic locations.

**Supplemental Table S3.** Location and function of gene variants associated with extrathoracic organ involvement.

| Gene | Position(hg19) | dbSNP | Location | Function |
| --- | --- | --- | --- | --- |
| PTGS2 | chr1:186645927 | rs2066826 | intronic |  |
| PACERR | chr1:186650751 | rs689466 | downstream |  |
| HLA-B | chr6:31323020 | rs2276448 | intronic |  |
| NOTCH4 | chr6:32192107 | rs3134929 | upstream |  |
| NOD2 | chr16:50744624 | rs2066842 | exonic | missense |
| NOD2 | chr16:50745199 | rs2066843 | exonic | synonymous |
| ITGAE | chr17:3637915 | rs220465 | intronic |  |
